# Dermatology associated with increased lichen sclerosus diagnosis and clobetasol use in rural health system

**DOI:** 10.1097/JW9.0000000000000254

**Published:** 2026-02-11

**Authors:** Hamna Khalid, Ruba Shaik, Anisah Alladeen, Ayesha Ahmad, Bryan McConomy

**Affiliations:** a Carle Illinois College of Medicine, University of Illinois Urbana-Champaign, Urbana, Illinois; b School of Medicine, Weill Cornell Medicine, New York, New York; c Boonshoft School of Medicine, Wright State University, Fairborn, Ohio; d Department of Hospital Medicine, Carle Foundation Hospital, Urbana, Illinois

**Keywords:** calcineurin inhibitors, clobetasol, diagnostic disparities, lichen sclerosus, rural health, vulvar dermatology

**What is known about this subject in regard to women and their families?**

- Lichen sclerosus (LS) is a chronic inflammatory skin condition that disproportionately affects postmenopausal women, often manifesting in the vulvar region with symptoms such as pain, itching, and scarring that can profoundly impact quality of life, sexual health, and emotional well-being.
- Despite its prevalence, LS is frequently misdiagnosed or undertreated, especially in areas where formal training in vulvar dermatology is limited.
- This diagnostic gap can lead to delays in treatment, exacerbating patient distress and placing additional burdens on families navigating fragmented care pathways.
- Dermatologists are significantly more likely to diagnose LS and prescribe evidence-based treatments like clobetasol and calcineurin inhibitors, whereas other specialties may underprescribe or omit therapy altogether.
- In rural health systems with limited dermatology access and long referral wait times, these disparities can worsen outcomes and prolong suffering.
- For women and their families, this means enduring symptoms without timely relief, facing uncertainty about diagnosis, and struggling to access appropriate care.
- Addressing these gaps requires empowering frontline providers with targeted education, clear treatment algorithms, and teledermatology support to ensure that all patients, regardless of where they first seek care, receive prompt, guideline-concordant management.

**What is new from this article as messages for women and their families?**

- This article offers new insights into how LS is diagnosed and treated across specialties, particularly in rural settings.
- It shows that dermatologists are more likely to diagnose LS and prescribe guideline-recommended treatments like clobetasol and calcineurin inhibitors.
- While Obstetrics and Gynecology and adult medicine providers also care for patients with vulvar complaints, they may be less likely to initiate these therapies, potentially due to limited exposure to vulvar dermatology during training rather than a lack of clinical skill.
- For families, this means that even when women seek care promptly, they may face delays in diagnosis or treatment unless referred to dermatology.
- In rural health systems, where dermatology providers are few and referral wait times can stretch for months, this bottleneck becomes especially problematic.
- Women may endure prolonged symptoms without relief and struggle to navigate lengthy care pathways.
- The study highlights how these delays in initiating appropriate therapy can worsen outcomes and increase the emotional and logistical burden on patients and caregivers.
- By identifying these patterns, the article underscores the need for clearer care pathways, better crossspecialty education, and systems-level solutions, such as teledermatology support and specialty-agnostic treatment algorithms, that empower frontline providers to recognize and treat LS at the first encounter.
- These changes are particularly urgent in rural communities, where access to specialty care is limited and timely intervention can make a significant difference in quality of life.

## To the Editor,

Lichen sclerosus (LS) is a chronic inflammatory dermatosis predominantly affecting the anogenital region of postmenopausal women.^1^ It presents as hypopigmented, atrophic plaques, and although its pathogenesis remains incompletely defined, with autoimmune mechanisms, hormonal factors, and local trauma implicated.^2^ Despite its prevalence, LS is often misdiagnosed or undertreated, leading to considerable morbidity and diminished quality of life.^2^

Educational gaps in LS management exist across specialties. A nationwide assessment showed training in inflammatory vulvovaginal skin disease is limited to dermatology programs.^3^ Gynecology trainees frequently report inadequate preparation for vulvar disorders, and over one-third of primary care clinicians report never having received education on vulvar skin disease.^4^ Since patients with genital complaints may first present to family medicine, obstetrics–gynecology, or adult medicine providers, the specialties most commonly diagnosing LS and their diagnostic and therapeutic approaches remain unclear.^5^

This study investigates which provider specialties most commonly diagnose LS, evaluates racial disparities in diagnosis, and characterizes treatment variations by specialty.

We conducted an Instiutional Review Board-exempt, retrospective cohort analysis of all International Classification of Diseases, Tenth Revision, Clinical Modification–coded LS diagnoses at the Carle Health System from 2015 to 2025 using Epic’s SlicerDicer feature. Patient age, self-reported race (White; Black/African American; Asian; American Indian/Alaskan Native; Native Hawaiian/Pacific Islander; all other; patient refused; and unknown), diagnosing provider specialty (dermatology, family medicine, Obstetrics and Gynecology (OB/GYN), adult medicine, and other), and topical treatment orders (nonclobetasol steroids, noncorticosteroid agents, calcineurin inhibitors, clobetasol, or other) was collected. Diagnostic odds ratios (ORs) with 95% confidence intervals (CIs) were calculated, and *χ*^2^ tests with Bonferroni-adjusted α = 0.0083 assessed racial and prescribing differences. Descriptive statistics summarized age distributions; 2-sided *P* ≤ .05 denoted significance.

Among 4,413 patients, over 90% identified as White across specialties, with no significant racial differences (overall *χ*^2^ = 9.49, *P* = .03; dermatology vs OB/GYN *χ*^2^ = 2.28, *P* = .32; vs adult medicine *χ*^2^ = 1.53, *P* = .47). Ages ranged from 7 to 103 years (mean 65–68 years by specialty) (Table 1).

**Table 1:** Patient cohort characteristics and odds ratio of lichen sclerosus diagnosis by specialty

Specialty	*n*	Age range (years)	Mean age	% White	Odds ratio (95% CI)	*Z*-score	*P*-value
Dermatology	1,128	7–99	67	>90%	–	–	–
OB/GYN	1,245	21–99	65	>90%	3.23 (2.70–3.85)	13.0	<.001
Family medicine	1,697	8–103	66	>90%	1.08 (0.88–1.32)	0.75	.48
Adult medicine	1,293	8–100	68	>90%	2.20 (1.85–2.62)	8.85	<.001
Other	50	17–99	71	>90%	–		–

Dermatology providers had higher odds of diagnosing LS than OB/GYN (OR 3.23, 95% CI [2.70–3.85], *P* < .001) and adult medicine (OR 2.20, 95% CI [1.85–2.62], *P* < .001); Family Medicine did not differ significantly (OR 1.08, 95% CI [0.88–1.32], *P* = .48). Dermatology also prescribed clobetasol most frequently (*P* < .0001) and used calcineurin inhibitors more often than OB/GYN (*χ*^2^ = 21.15, *P* < .0001); rates of nonclobetasol steroids and no prescription were similar across specialties (Table 2).

**Table 2:** Prescription orders and statistical comparisons by specialty

Prescription category	Total orders	Dermatology versus OB/GYN (χ^2^; *P*)	Dermatology versus family medicine (χ^2^; *P*)	Dermatology versus adult medicine (χ^2^; *P*)	Dermatology versus all others (*P*)
Overall prescribing pattern	–	14.01; .0029	17.88; .0005	7.41; .059	–
Topical nonclobetasol steroids	3,039	NS	NS	NS	NS
Calcineurin inhibitors	812	21.15; <.0001	NS	NS	–
Clobetasol	4,480	<.0001	<.0001	<.0001	<.0001
None of the above	3,850	–	–	–	–

NS, not significant.

These patterns suggest that established referral pathways may concentrate LS cases in dermatology. A contributing factor may be limited exposure and insufficient training in diagnosing LS in specialties other than dermatology. Dermatologists’ markedly higher use of super-potent clobetasol and steroid-sparing calcineurin inhibitors aligns with evidence-based guidelines but also exposes potential under-recognition and undertreatment in primary care and OB/GYN settings. In a rural health system—with few dermatology providers and referral wait times often extending for months—this bottleneck may exacerbate delays in initiating appropriate therapy and worsen patient outcomes.

Future efforts should empower frontline clinicians to recognize anogenital LS and start guideline-concordant treatment at the first encounter. Tailored training modules for family medicine, OB/GYN, and internal medicine can strengthen confidence in diagnosing vulvar LS and prescribing super-potent topical corticosteroids. The adoption of clear, specialty-agnostic care algorithms and teledermatology support can mitigate the impact of provider shortages and reduce wait times for specialist input. Prospective studies monitoring treatment adherence, symptom improvement, and patient-reported outcomes across care settings will be key to refining management protocols and closing treatment gaps.

Limitations include retrospective design, reliance on coded electronic health record data prone to miscoding or missing information, absence of histopathologic confirmation, single-institution scope, and aggregate-level analysis precluding individual-level insights. In addition, clobetasol was the only class I corticosteroid independently assessed; other equivalent agents were grouped under nonclobetasol steroids and not individually evaluated, representing a further limitation.

## Conflicts of interest

None.

## Funding

None.

## Study approval

Because all data was derived from the fully deidentified Epic SlicerDicer database, this study was determined to be nonhuman subjects research by the Carle Institutional Review Board.

## Author contributions

HK: Participated in the research design, conceptualized the study, and performed the primary data analysis. RS: Contributed to the data analysis and assisted in the interpretation of results. A Alladeen and A Ahmad: Participated in the writing and revision of the manuscript and contributed to the intellectual development of the work. BS: Served as the senior author and principal investigator, overseeing the study design, providing critical revisions, and ensuring the integrity and accuracy of the analysis. All authors reviewed the final manuscript, approved its submission, and agreed to take public responsibility for its content.

## References

[1] De Luca DA Papara C Vorobyev A. Lichen sclerosus: the 2023 update. Front Med 2023;10:1106318. 10.3389/fmed.2023.1106318 PMC9978401 36873861

[2] Ventolini G Patel R Vasquez R. Lichen sclerosus: a potpourri of misdiagnosed cases based on atypical clinical presentations. Int J Womens Health 2015;7:511–5. 26056492 10.2147/IJWH.S82879 PMC4431497

[3] Comstock JR Endo JO Kornik RI. Adequacy of dermatology and OB-GYN graduate medical education for inflammatory vulvovaginal skin disease: a nationwide needs assessment survey. Int J Womens Dermatol 2020;6:182–5. 32637541 10.1016/j.ijwd.2020.01.008 PMC7330429

[4] Jennifer Y Catrina C Donna M Rachel P. Who should manage vulvar disorders? A national survey of gynecology and dermatology residents [16H]. Obstet Gynecol 2017;129:86S.

[5] Crew A Leatherland R Clarke L Owen C Simpson RC. Barriers to diagnosing and treating vulval lichen sclerosus: a survey study. Br J Gen Pract 2025;75:e250–6. 39516015 10.3399/BJGP.2024.0360 PMC11881009

